# Inulin alleviates offspring asthma by altering maternal intestinal microbiome composition to increase short-chain fatty acids

**DOI:** 10.1371/journal.pone.0283105

**Published:** 2023-04-04

**Authors:** Guifang Yuan, Song Wen, Xuemei Zhong, Xiaotong Yang, Linrui Xie, Xiuli Wu, Xiaoyu Li

**Affiliations:** 1 Department of the First Clinical Medicine, Chongqing Medical University, Chongqing, China; 2 Familial & Hereditary Cancer Center, Key Laboratory of Carcinogenesis and Translational Research (Ministry of Education), Peking University Cancer Hospital & Institute, Beijing, China; 3 Department of Respiratory Endocrinology, School of Clinical Medicine, Chongqing Medical and Pharmaceutical College, Chongqing, China; 4 Department of the 2nd Clinical Medicine, Chongqing Medical University, Chongqing, China; 5 The Ninth People’s Hospital of Chongqing, Chongqing, China; 6 Laboratory of Innovation, Basic Medical Experimental Teaching Centre, Chongqing Medical University, Chongqing, China; Universite du Quebec a Montreal, CANADA

## Abstract

Technically, symptom of offspring asthma is also closely reliant on its maternal high-fiber diet as well as the intestinal microbiome. Fruits and vegetables are abundant in inulin, and this naturally soluble dietary fiber is endowed with a potential value on offspring asthma control through the maternal intake, but the mechanism now remains less studied. In this study, rats were given with inulin-included drinking water, whereas in normal group rats were allowed with normal water. Afterwards, we analyzed both the formations of the offspring intestinal microbiome ahead of asthma model establishment and of the maternal intestinal microbiome through high throughput sequence and the short-chain fatty acids (SCFAs) by metabolomic analysis. Subsequently, lung inflammation indexes were detected by Elisa, and the expression of short-chain fatty acid receptors (GPR41, GPR43) in the offspring of asthma models were evaluated through qPCR assay. Inulin intake resulted in altered maternal intestinal microbiome composition, with a significant increase in SCFAs-producing bacteria (mainly *Bifidobacterium*), attenuating the asthmatic inflammatory response in the offspring. Meanwhile, inulin intake during pregnancy modulates the composition of the intestinal microbiome of the offspring, and this alteration appears before the onset of asthma, hence, there should be further studies onto the impacts of offspring’s intestinal microbiome on asthma procession.

## Introduction

Asthma is a chronic inflammatory disease of the airways featured with the main pathophysiological change of airway hyperresponsiveness (AHR) [1]. Epidemiological studies over the past decades found the asthma prevalence in children notably inflated, and it was becoming a serious problem jeopardizing public health [2]. It was recently reported that high-fiber diets and intestinal microbiome are possible factors in relation to asthma pathology. Diets of the intakes that are high in fat and low in fiber increase asthma risk and probably eventually end up in worsening airway inflammation and lung function in asthmatics; reversely, intakes of high fiber and low fat like fruits and vegetables decrease asthma risk [3, 4]. Herbst et al. [5] found the effects of intestinal microbiome on allergic airway inflammation, for high-fiber diets affect not only the pathology of allergic asthma by altering intestinal microbiome composition but also the intestinal microbiome metabolite like short-chain fatty acids (SCFAs) [6].

Inulin, one of the soluble dietary fibers widely found in fruits and vegetables, extracted mainly from the monocotyledonous and dicotyledonous families. And chicory is rich in inulin, and it is an ideal source for conventional extraction [7]. Intake of inulin brings forth alterations in intestinal microbiome composition, resulting impacts on *Bifidobacterium* and *Lactobacillus* in varying degrees, by which it is deemed as a wide prebiotic [8–10]. Some studies have revealed inulin’ capacity in magnifying intestinal beneficial floras and escalating the production of the metabolites like short-chain fatty acids, so that the potent anti-tumor effects through immune modulation were established [11]. Preliminary clinical trials have elucidated the improvement of oral inulin supplementation in alleviating adult asthmatics’ respiratory inflammation in manners of benefiting the asthma control and modulating intestinal microbiome, which suggests the inulin’s role as an adjunct in asthma treatment in a possible means of short-chain fatty acids [12].

Short-chain fatty acids (acetic acid, propionic acid and butyric acid) are mainly produced by the bacteria in the colon that ferment dietary fiber [13, 14]. It has been shown that SCFAs can regulate body metabolism, can prevent obesity, regulate glucose and lipid metabolism as well as influence insulin [15]. In addition, SCFAs play an important role in immunity, inflammation, and tumors [16]. SCFAs exert their anti-inflammatory effects mainly through the G protein-coupled receptors (GPCRs) activation pathway (mainly GPR41, GPR43) and Histone deacetylases (HDACs) inhibition pathway [17]. Of all the SCFAs, butyrate is endowed with the potential to treat allergic asthma by activating immune cells [18]. Maslowski et al. [19] found that stimulation of GPR43 by SCFAs functions as a manipulator in the control of colitis, arthritis and asthma. Aurélien Trompette et al. [20] showed that a high-fiber diet increases the levels of SCFAs and reduces the severity of pulmonary allergic inflammation by shaping the immune environment in the lungs, where the effect of propionate on allergic inflammation is dependent on GPR41 rather than GPR43. In vitro studies by Mizuta Kentaro found GPR41 expression in human airway smooth muscle cells, highlighting SCFAs’ possible direct efficiency on the bronchial cells [21].

Asthma susceptibility is likely determined early in life for a large majority of patients [22]. The impact of maternal diet during pregnancy on the fetus and future child health is a concerned question [23]. Many studies have demonstrated that offspring development and health are associated with maternal high-fiber diet and intestinal microbiome [24, 25]. Maternal intestinal microbiome drives innate immune development in the early postnatal period of the offspring, and is important in regulating neural, peripheral and intestinal immune development of the embryo [26, 27]. Russell et al. [28] found that vancomycin intervention in perinatal and neonatal mice increased their susceptibility to asthma later in life. One study found that SCFAs produced by maternal intestinal microbiome were able to cross the placental barrier to modulate GPR43 and GPR41 in sympathetic, intestinal epithelial and pancreatic B cells of offspring mice making them resistant to obesity in adulthood [29]. Alison N. Thorburn’s study showed that feeding gestating mice a high-fiber diet significantly reduced offspring susceptibility to allergic airway disease, and that this effect came from SCFAs, the fermentation products of intestinal microbes [30]. However, the specific mechanism by which inulin, a dietary fiber, modulates asthma in offspring and links maternal and offspring health is unknown.

## Materials and methods

### Animals

This study was undertaken with the Ethics Committee of Chongqing Medical University Approval Committee. All surgery was performed under urethane anesthesia, and all efforts were made to minimize suffering. 14 SPF female Sprague-Dawley (SD) rats and 28 male SD rats, 9w, were provided by the Experimental Animal Center of Chongqing Medical University [SCXK-(Chongqing) 2018–0003]. All experimental protocols were performed according to the guidelines for the care and use of laboratory Animals, and they were approved by the Institutional Ethics Committee of Chongqing Medical University. After one week of adaptive feeding under standard conditions, each female rat was mated overnight with 2 males. The morning of vaginal plug detection was defined as embryonic day (E) 0.5 (E0.5). The 14 female rats were equally divided into two groups: normal maternal group (NM) and inulin maternal group (IM). Starting at E12.5, the IM group was fed with drinking water containing 10% inulin (Sangon Biotech Co., Ltd. Shanghai, China) for a week, while the NM group was fed with purified water. At E18.5, collecting feces from two groups of maternal animals separately. During the experiment, one rat in NM died, and another had stillbirths. None of them were supplemented.

The offspring of the two groups were recorded as normal offspring group (NO) and inulin offspring group (IO), 16 offspring each, half male and half female. Feces were collected when the offspring were 3 weeks old, and then asthma molds were performed. On days 1 and 8, all offspring were injected with 1 mg of OVA and 200 mg of Al (OH)3, which were emulsified in aseptic saline in a total volume of 1 ml. In particular, 0.4 ml of the emulsified treatments was intraperitoneally injected, 0.2 ml was injected to the two anterior toes, and 0.4 ml was injected to the bilateral groins. On day 15, all of the offspring were exposed to aerosolized grade II OVA (1% wt/vol diluted in a saline solution) for 30 min daily for 2 weeks. Grade II and V OVA were purchased from Sigma (MO, USA). Al(OH)_3_ was acquired from Aladdin (Shanghai, China). The tissues were collected within 24h after the end of nebulization on the last day. The rats were anesthetized by inhaling 3 Vol% isoflurane (Veteasy® 100%(V/V), RWD life science, Shenzhen, Guangdong Rrovince, China) in a chamber for 5 min and maintained anesthesia at 1.5 Vol% isoflurane with 0.8L/min air as the carrier via a nose cone. Air pump (R510-25, RWD life science) was used as an air source throughout the anesthesia. Alcohol (75%) was used to sterilize the skin on the chest and abdomen of the rats. After blood was collected by cardiac puncture, they were sacrificed by cervical dislocation and then dissected under aseptic conditions. The left lobes were used for qPCR assay, and the lower right lobes were preserved in 10% formalin solution for hematoxylin and eosin (H&E) staining. The blood was centrifuged at 3,000 rpm at 4°C for 10 min, and the serum was collected and stored at −80°C until analysis.

### Determination of IgE, IL-4, INF-γ, IL-17 levels, and H&E staining in offspring

Enzyme-linked immunosorbent assay (ELISA) kits were used to determine IgE, IL-4, INF-γ and IL-17 levels (Ruixin Biotechnology Co., Ltd. Quanzhou, China), and the kit instructions were; ‘take out the serum samples from the refrigerator and gradually return to room temperature, and then measure the serum IgE, IL-4, INF-γ and IL-17 levels’. After fixing the lung tissue with 10% formalin solution for 24 hours, it was embedded in paraffin, sectioned (3μm), and stained with HE. The infiltration of inflammatory cells in the lungs of rats was observed under microscope.

### Short chain fatty acids extraction and analysis in maternal rats

Place an approximately 50 mg sample of feces into a 2 ml grinding tube, add a steel ball, 450 μl of methanol, and 50 μl of internal standard (1000 μg/ml of 2-ethyl-butyric acid, methanol configuration), and grind it in a freezing grinder 50HZ 3 twice per minute. Then ultrasonic the sample in an ice-water bath for 30 minutes, stand at -20°C for 30 minutes, and centrifuge at 13000g for 15 minutes (4°C). Transfer the supernatant to a 1.5 ml centrifuge tube. Add 50mg of anhydrous sodium sulfate, vortex, centrifuge at 13000g for 15min (4°C) and take the supernatant solution on the machine for gas chromatographic analysis. The analytical instrument used was Agilent Technologies Inc. (CA, USA) 8890B-5977B GC/MSD GC/MSD. HP FFAP capillary column (30 m × 0.25 mm × 0.25 μm, Agilent J&W Scientific, Folsom, CA, USA). The protocol used included a carrier gas that was high-purity helium (purity not less than 99.999%), the flow rate was 1.0 ml/min, the inlet temperature is 260°C, an injection volume of 1μl, split injection, split ratio 10:1, and solvent extension 3min. Program temperature rise: the initial temperature of the column oven is 80°C, the temperature was programmed to increase to 120°C at 40°C/min increments, and then increased to 200°C at 10°C/min steps, and finally runs at 230°C for 6 minutes. Mass spectra were collected by using an electron impact ion source (EI), ion source temperature 230°C, quadrupole temperature 150°C, transmission line temperature 230°C, and electron energy 70eV. The scanning mode is the full scan mode (SCAN), and the quality scan range: m/z: 30–300. The obtained data were assessed by using Masshunter quantitative software (Agilent, USA, version number: v10.0.707.0) to automatically identify and integrate each ion fragment with default parameters and assist manual inspection. Linear regression standard curve lines were drawn with the mass spectrum peak area of the analyte as the ordinate and the concentration of the analyte as the abscissa. Sample concentrations were calculated by substituting the mass spectrum peak area of the sample analyte into the linear equation to calculate the concentration results.

### qPCR detection on GPR41 and GPR43 in offspring lung tissue

14 SPF Total RNA was extracted with the RNAiso Plus (Takara, Tokyo, Japan). cDNA was reverse transcribed using isolated RNA samples as templates and Goldenstar™ RT6 cDNA Synthesis Kit (Tsingke Biotechnology Co., Ltd. Beijing, China). Expression analyses were performed via 2 ×T5 Fast qPCR Mix (SYBR Green I) (Tsingke Biotechnology Co., Ltd. Beijing, China) and the Applied Biosystems™ 7500 Real-Time PCR System. Real-time PCR cycling conditions were set up as follows: 95°C for 30 s, followed by 40 cycles of 95°C for 5 s, 55°C for 30 s, and 72°C for 30 s. The GAPDH gene was used as an internal control. Each sample was tested in duplicate for the average Ct value. Relative mRNA expression was calculated after normalization to the GAPDH reference gene using the 2-DDCt method. The primer sequences of the genes were showed in Table 1.

**Table 1:** Primer sequences used for qPCR.

Gene		Primer Sequences (5′-3′)
**GPR43**	**Forward**	**CAATCCCGGCTCCTCTATGC**
	**Reverse**	**TTTGACTCCCACCCCTGTCT**
**GPR41**	**Forward**	**TATGTGGCGCAGAAGACGAG**
	**Reverse**	**TGACATCTGACTGCTCCGTG**
**GAPDH**	**Forward**	**GCAAGTTCAACGGCACAG**
	**Reverse**	**GCCAGTAGACTCCACGACATA**

### DNA extraction, PCR amplification

The frozen fecal samples were thawed at room temperature and then homogenized via bead beating (FastPrep bead matrix E, MP Biomedicals, Santa Ana, CA, USA) with 500 μl of aseptic saline. Aseptic saline was added until a volume of 1000 μl was obtained, and bacterial DNA was extracted with a bacterial genomic DNA extraction kit as per the manufacturer’s instructions. After DNA concentration was determined, 1 μl of DNA was diluted to 100 ng/μl with ultra-pure water and then stored at −20°C.

Universal primers 338F/806R were synthesized to amplify the 16s rDNA V4–V5 region. The sequences of the forward primer and reversed primer were 5′-ACTCCTACGGGAGGCAGCAG-3′ and 5′-GGACTACHVGGGTWTCTAAT-3′, respectively. In brief, PCR consisted of 4 μl of 5× FastPfu Buffer, 2 μl of dNTPs (2.5 mM), 0.8 μl of forwarding primer (5μM), 0.8 μl of reverse primer (5 μM), 0.4 μl of FastPfu polymerase, 10 ng of template DNA, and ultra-pure water added to obtain a volume of 20 μl. The reaction conditions were as follows: 5 min at 95°C, 27 cycles of 30 s each at 95°C and 53°C and 45 s at 72°C, and 10 min at 72°C.

### Metagenomic sequencing and data analysis

DNA extract was fragmented to an average size of about 400 bp using Covaris M220 (Gene Company Limited, China) for paired-end library construction. Paired-end library was constructed using NEXTFLEX Rapid DNA-Seq (Bioo Scientific, Austin, TX, USA). Adapters containing the full complement of sequencing primer hybridization sites were ligated to the blunt-end of fragments. Paired-end sequencing was performed on Illumina Novaseq 6000 (Illumina Inc., San Diego, CA, USA) at Majorbio Bio-Pharm Technology Co., Ltd. (Shanghai, China) using NovaSeq Reagent Kits according to the manufacturer’s instructions (www.illumina.com).

The data were analyzed on the free online platform of Majorbio Cloud Platform (www.majorbio.com). Briefly, the paired-end Illumina reads were trimmed of adaptors, and low-quality reads (length<50 bp or with a quality value <20 or having N bases) were removed by fastp (https://github.com/OpenGene/fastp, version 0.20.0).

### Statistical analysis

The Prism software (GraphPad) was used for statistical analysis of the data. Student’t test was applied to two datasets conforming to a normal distribution and homogeneity of variance. Welch’s t test was used when variances are unequal between groups. (*, p <0.05; **, p <0 .01; ***, p <0 .001). The Wilcoxon rank-sum test was performed to analysis the inter-group differences in the flora composition.

## Results

### Indicators of inflammation in the offspring

**Fig 1:**
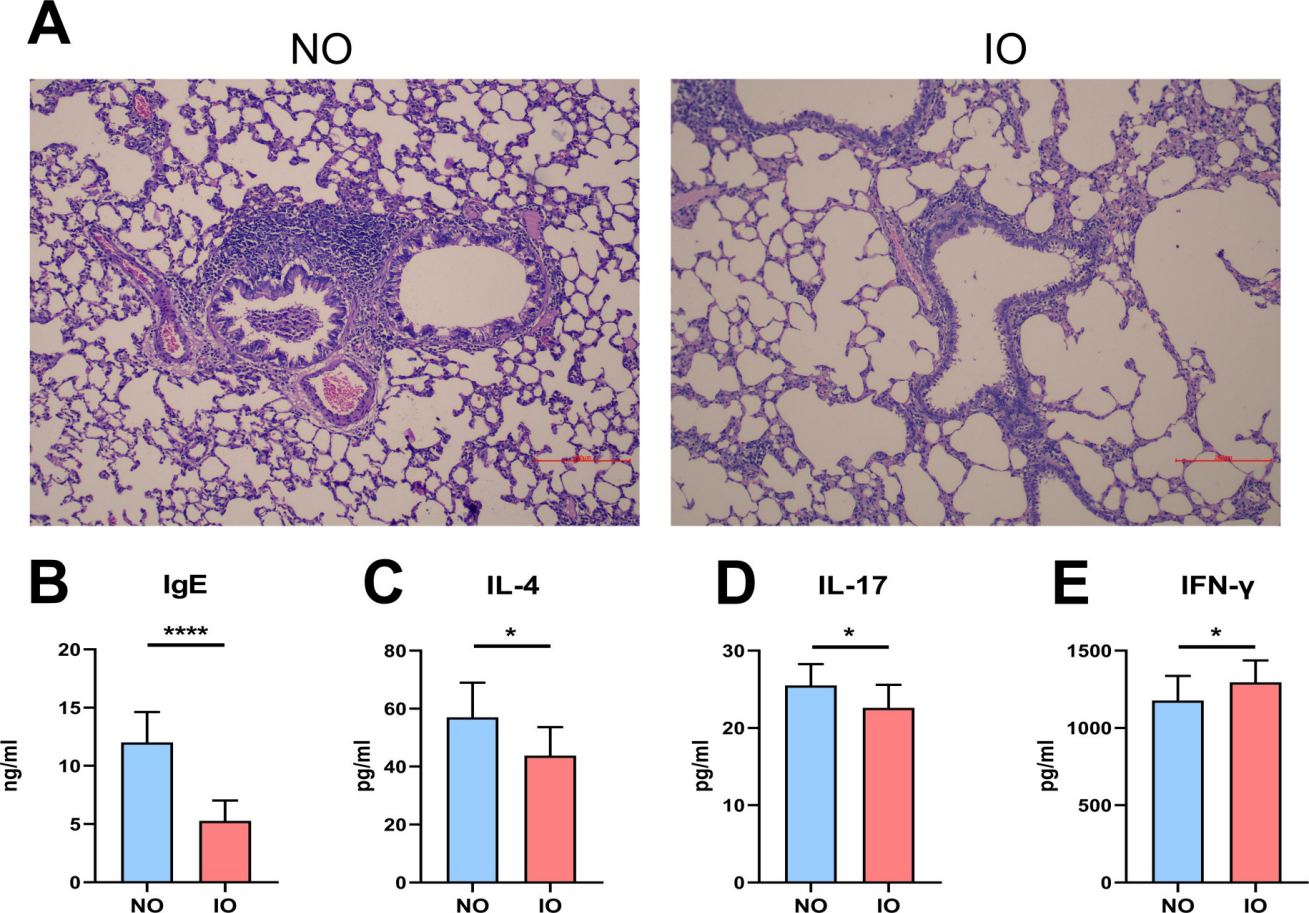
Inulin is able to reduce lung inflammation and related cytokine production in the offspring. (A) The pulmonary inflammation in NO group was significantly worse than that in IO group; The level of IgE (B), IL-4 (C) and IL-17 (D) in NO group was higher than that in IO group (B: p < 0.0001, C: p <0.05, D: p <0.05); (E) The INF-γ level in IO group was higher than that in NO group (p <0.05).

Pathological sections of the lung showed that when compared with the NO group, and the IO group had a relatively complete airway epithelial structure, and the degree of inflammatory cell infiltration around the bronchus and blood vessels was significantly reduced. Exudates in the lung interstitium and alveolar cavity were also significantly reduced (Fig 1A). The levels of IgE, IL-4 and IL-17 in IO group were statistically significantly higher than those in NO group, but the levels of INF-γ in IO group were significantly lower than those in NO group, and the results were independent of gender (Fig 1B–1E).

### Analysis on short-chain fatty acid (SCFAs)

**Fig 2:**
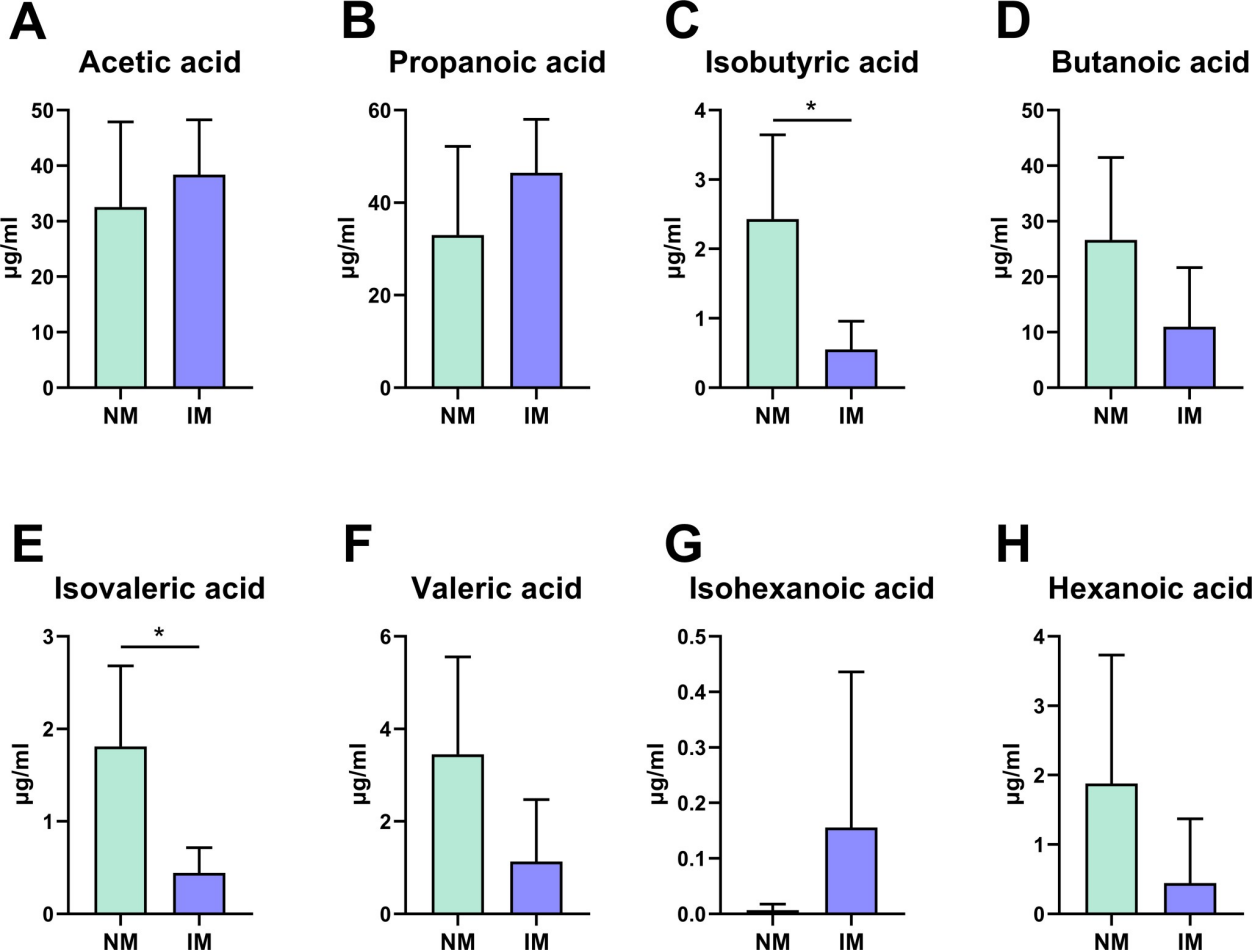
Analysis of short-chain fatty acid content in feces of maternal rats. (A-H) Quantitative analysis on short-chain fatty acids in maternal feces at E18.5.

Inulin’s efficacy on human health and disease are accomplished through the path of the production of short-chain fatty acids in the intestine. We used GC-MS to detect the SCFAs content in feces of maternal rats (Fig 2). Surprisingly, the levels of various SCFAs in the inulin maternal group were lower than those in the normal maternal group except for Acetic acid, Propionic acid and Isohexanoic acid. Among them, Isobutyric acid and Isovaleric acid were statistically significant.

### Expression of GPR41 and GPR43 in the lungs of offspring

**Fig 3:**
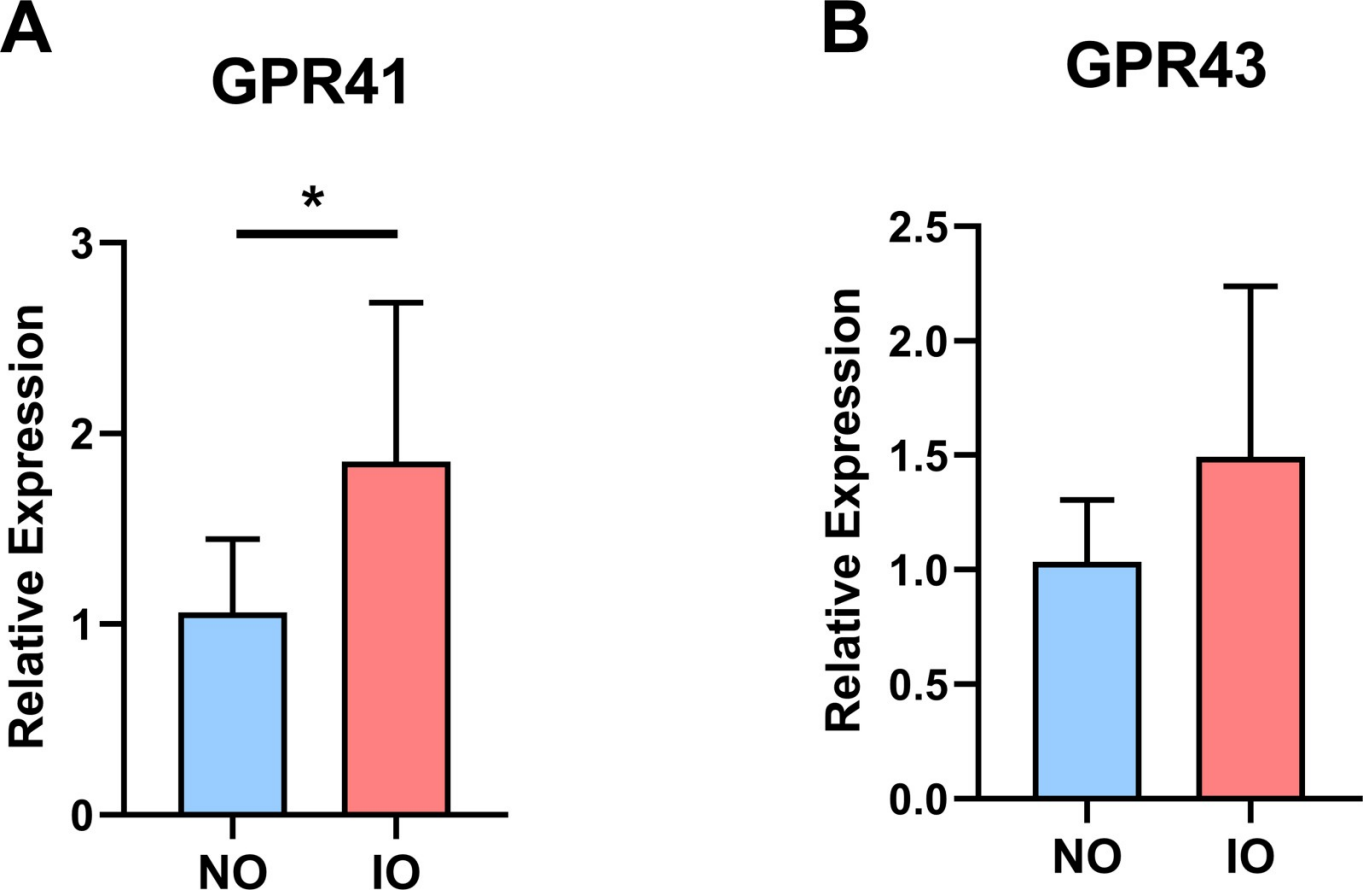
Expression of GPR41 and GPR43 in the lungs of offspring in the asthma model. (A) The relative expression of GPR41 mRNA in the lungs of the IO group was higher than that of the NO group (p <0.05). (B) The relative expression of GPR43 mRNA in the lungs of the IO group was higher than that of the NO group (p >0.05).

To further investigate the relationship between short-chain fatty acids and susceptibility to allergic asthma in offspring rats, the relative expressions of GPR41 and GPR43, the two short-chain fatty acid receptors, were detected by qPCR in the lung tissues of inulin offspring group and normal offspring group after asthma modeling (Fig 3). We were surprised to find that the relative mRNA expressions of GPR41 and GPR43 were higher in the offspring of the inulin maternal group than in the offspring of the normal group, with GPR41 being statistically significant.

### Microbial diversity analysis

Short-chain fatty acids are metabolites of dietary fiber produced by intestinal flora. Since the results of the short-chain fatty acid assay and the relative expression of the mRNA of its receptor were not consistent, further analysis of the microbial diversity of maternal rats and offspring was performed.

#### Alpha-diversity

The student`s T-Test analysis was applied to bacterial DNA and genomic data. The Shannon index, Simpson index, and Chao index were shown in Table 2. The results showed that the Shannon index of the IM and OM groups were significantly different (p <0.05), however no difference in the Simpson indexes for each group was found. This indicates that there was a difference in community diversity between the IM and OM groups. The Chao indexes indicated the significant differences between the IM and OM groups (p <0.05). These results confirmed that there were significant differences in community richness between IM group and those in no soluble fiber diets.

**Table 2:** The alpha diversity analysis of microbiome in feces of maternal rats and offspring.

Group		CHAO	SHANNON	SIMPSON
**Maternal rats**	**IM**_**mean±sd**_	**247.8571±200.8170**	**3.2071±0.6875**	**0.1152±0.0793**
	**NM**_**mean±sd**_	**611.6000±189.0233**	**4.1713±0.6331**	**0.0543±0.0241**
	**P**_**IM-NM**_	**0.0101**	**0.0330**	**0.1316**
**Offspring**	**IO**_**mean±sd**_	**390.6250±228.6688**	**3.1634±0.6472**	**0.1228±0.0608**
	**NO**_**mean±sd**_	**395.3750±256.6643**	**3.0361±0.3754**	**0.1115±0.0509**
	**P**_**IO-NO**_	**0.9694**	**0.6379**	**0.6914**

#### Community-composition and beta-diversity

**Fig 4:**
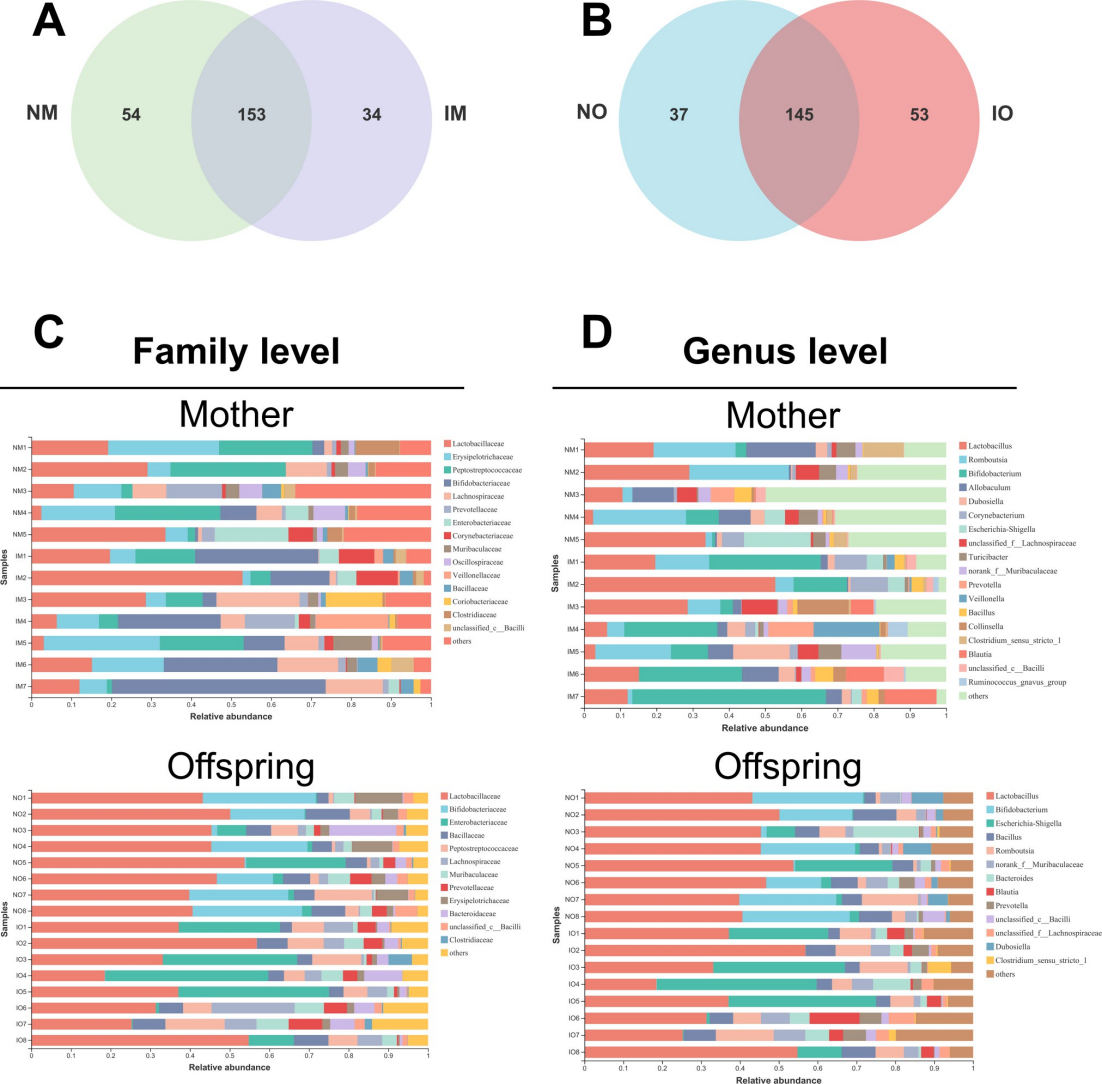
Fecal microbial composition analysis on maternal rats and offspring. (A, B) Shared ASV analysis of the maternal and offspring groups. Venn diagram revealed the unique and shared ASVs in the different groups; (C, D) Analysis on the fecal microbial composition of the maternal and offspring groups on the family level and genus level.

The total and particular ASV sequences in the four groups were calculated with the R programming language (Fig 4A and 4B). A total of 241 ASVs were shared by the maternal groups. A total of 54 and 34 particular ASVs were present in NM and IM, respectively. A total of 235 ASVs were shared by offspring groups. A total of 37 and 53 particular ASVs were present in NO and IO, respectively. This finding indicated that the number of specific ASVs is higher in those maternal rats fed with inulin than in normal rats, while the opposite is true for their offspring.

Community barplot analysis showed that on the family level (Fig 4C), the dominant families in NM group were *Lactobacillaceae*, *Peptostreptococcaceae*, and *Erysipelotrichaceae*; the dominant families in IM group were *Bifidobacteriaceae*, *Lactobacillaceae*, and *Erysipelotrichaceae*; The abundance of *Bifidobacteriaceae* in IM group increased significantly, resulting in the emergence of a newly dominant family of bacteria. And the dominant families in NO group were *Lactobacillaceae*, and *Bifidobacteriaceae*; while the dominant families in IO group were *Lactobacillaceae*, and *Enterobacteriaceae*.

At the Genus level (Fig 4D), the proportion of *Bifidobacterium* was significantly higher in IM group when compared to the NM group and it was the predominant dominant genus. Additionally, some SCFAs-producing genera were also increased in IM group, such as *Lactobacillus*, *Blautia*, and *Prevotella*. While in the offspring, the dominant genera in NO group were *Lactobacillus*, and *Bifidobacterium*; the dominant genera in IO group were Lactobacillus, and *Escherichia-Shigella*. The proportion of *Lactobacillus*, and *Bifidobacterium* decreased in IO group, while the proportion of *Escherichia-Shigella* was higher.

**Fig 5:**
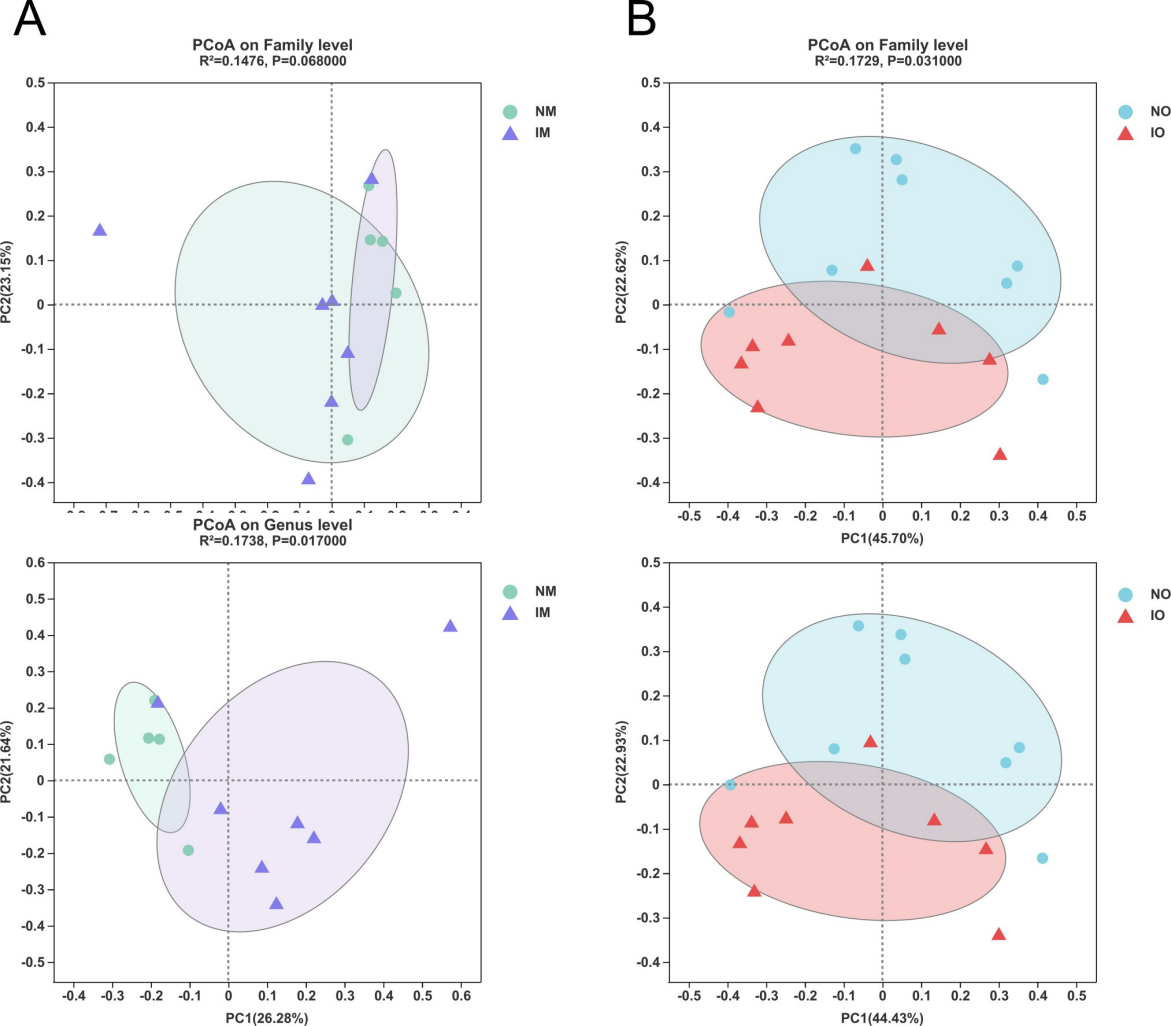
Fecal microbial composition analysis and beta-diversity analysis on maternal rats and offspring. (A, B) PCoA analysis was to explore the correlation of the composition of the microbiome on the family level and genus level of each group.

PCoA analysis and Adonis methods were to analyze bacterial populations at the family and genus levels, respectively (Fig 5). The PCoA analysis showed that at both the family level and the genus level, the microbiome composition in the NM and IM groups overlapped but there was a tendency for the microbiome of the two groups of maternal rats to aggregate. Adonis results showed that at the family level, the difference between the two groups was not significant (R2 = 0.1476, p = 0.0680); at the genus level, the difference between the two groups was significant (R2 = 0.1738, P = 0.0170). And there were significant differences between the two groups of offspring at both family level and genus level (R2 = 0.1729, P = 0.0310; R2 = 0.1787, P = 0.0250). These data show that two offspring groups had statistically significant differences, irrespective of the scale of analysis (at family level or at genus level). But two maternal groups have statistically significant differences at only genus level.

#### Analysis on significant differences between the groups at genus levels

**Fig 6:**
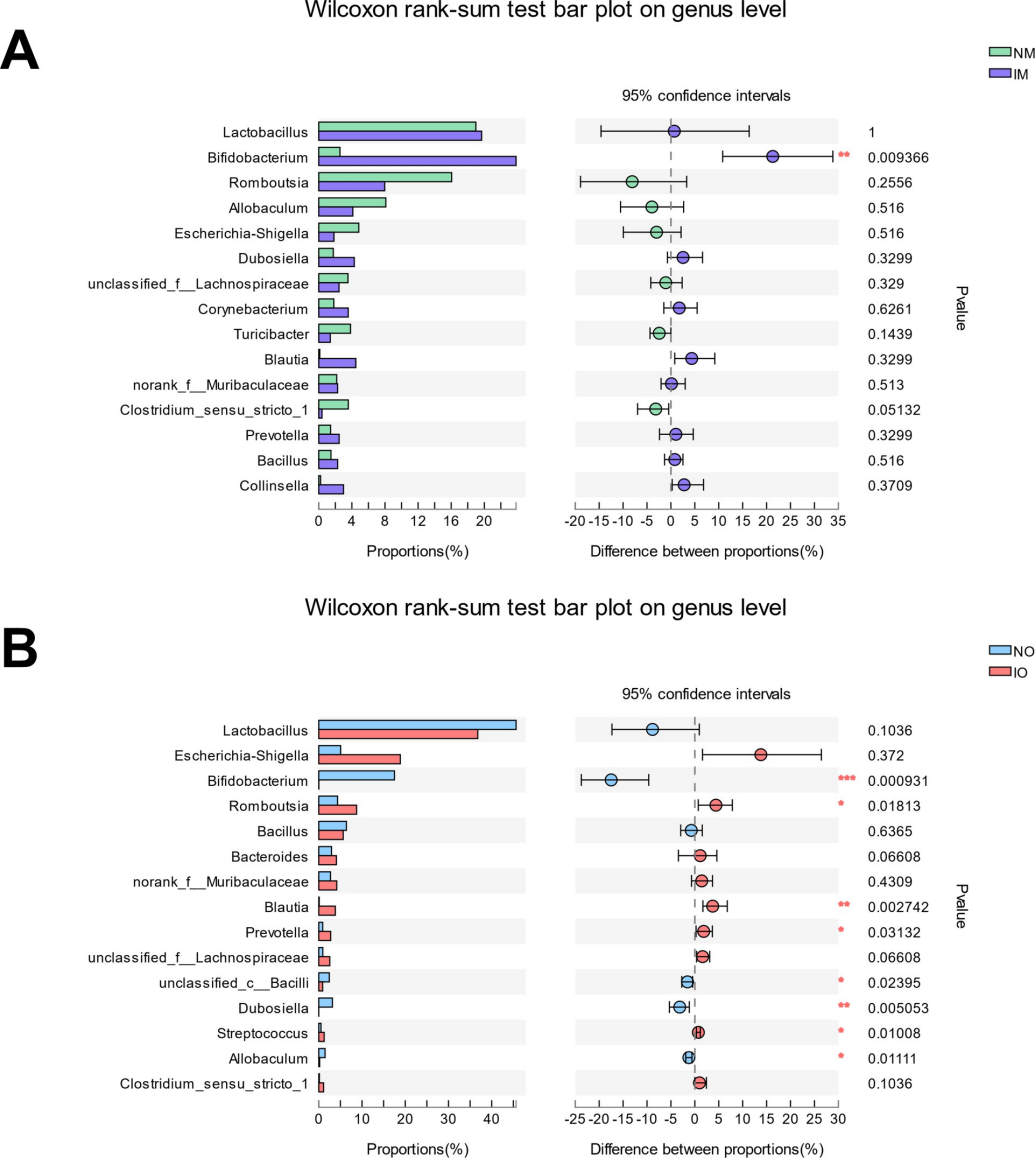
Analysis on the significantly different species at genus level. (A) Analysis on the significantly different species in maternal group; (B) Analysis on the significantly different species in offspring (*, p <0.05; **, p <0 .01; ***, p <0 .001).

The Wilcoxon rank-sum test was then applied to screen for key bacteria that may be associated with the development of asthma in offspring (Fig 6). Between the two groups of maternal rats, there was a highly significant difference in *Bifidobacterium* (p <0.01). And between those two groups of offspring, significant differences were found amid *Bifidobacterium*, *Romboutsia*, *Blautia*, *Prevotella*, *unclassified_c__Bacilli*, *Dubosiella*, *Streptococcus*, and *Allobaculum*.

## Discussion

Dietary fiber is an important nutrient, and it is found in close relation with asthma. High-fiber not only reduces the risk of asthma, but also extends life expectancy [3, 31]. It was reported that the adult offspring born to those pregnant rats that were fed with high-fiber feeding were imparted with robust resistance against severe allergic airways disease, which might be attributed to the production of short-chain fatty acids in the maternal intestine [29, 30]. In this study, pregnant rats in inulin group during gestation was fed with the aqueous solution containing 10% inulin; then the consequence of maternal inulin intake on lung inflammation was explored through the modeled asthma offspring from NM group and IM group. Results of Elisa revealed that there was a statistically significant decrease in INF-γ expression and an increase in IL-4, IgE, IL-17 levels, which suggesting that inulin intake during pregnancy improves lung inflammation in offspring with asthma, and this effect that is independent of gender. Th1/Th2 imbalance is the main mechanism of asthma. INF-γ, a representative of Th1 cytokines, inhibits inflammation, whereas IL-4, a representative of Th2 cytokines, promotes inflammation [32]. In the current study, the serum IgE concentration was also increased significantly. This finding was attributed to IL-4 promoting the secretion of B plasma cells. Ig E triggers mast cells to secrete histamine. IL-17 is an important cytokine that participates in allergic reactions and stimulate airway inflammation [1]. In the current study, H&E staining demonstrated numerous inflammatory cells infiltrating the lungs.

High-throughput sequences showed that the intake of inulin reduced the alpha diversity of the intestinal microbiome and decreased the total ASV number of intestinal bacteria in maternal rats, which was consistent with the findings of Song X’s study [8]. By further analysis of the sequencing data, we found significant differences in the composition of the intestinal microbiome in two groups of pregnant rats. At the genus level, the proportion of *Bifidobacterium* was significantly higher in NM group and became a new dominant genus, while it has been widely confirmed and recognized that inulin increases the number of *Bifidobacterium* in the intestinal microbiome [8, 10, 33].

Short-chain fatty acids are the major end products of carbohydrate metabolism in *Bifidobacterium* [34]. In addition to *Bifidobacterium*, the proportion of short-chain fatty acid-producing genera such as *Lactobacillus* [35], *Blautia* [36], and *Prevotella* [37] was also increased, but not statistically significant; PCoA analysis combined with Adonis analysis showed that difference between the two groups was significant at the genus level. It is worth pondering that in our results, short-chain fatty acids were reduced in the feces of IM group, which contradicts the results of the GPR41 assay in the lung tissue of the offspring as well as the results of the microbial composition analysis of the pregnant rats. It is possible that SCFAs were rapidly consumed by large bowel microbiota and colonocytes, and accordingly, their concentrations drop substantially between the cecum and the anus [38], which were consistent with Peter J Vuillermin et al [39].

Dietary fiber provided with the diet during pregnancy plays a key role in shaping the maternal intestinal microbiome [40]. In addition, some research reported that the maternal diet during pregnancy also can affect the offspring intestinal microbiome [41]. Therefore, we further analyzed the offspring intestinal microbiome and found that IO group produced more the total ASV number of intestinal bacteria. There was no difference in α-diversity between those two offspring groups, but the community composition was altered.

Unlike the Maternal group, the IO group had a significantly lower proportion of *Bifidobacteria* than the NO group, which is consistent with the results of the epidemiological experiment by Sara N Lundgren et al. [42]. In addition, we found a decrease in *unclassified_c__Bacilli*, *Dubosiella*, *Allobaculum* and other genera in IO group and an increase in *Romboutsia*, *Blautia*, *Prevotella*, *Streptococcus*, *Parabacteroides*, *Phascolarctobacterium* and other genera increased. Among them, *Bifidobacterium*, *Romboutsia*, *Blautia*, *Prevotella*, and *Phascolarctobacterium* are thought to produce short-chain fatty acids, which exert anti-inflammatory effects [43, 44]. Although the increase or decrease of different short-chain fatty acid producing bacteria was not completely consistent between the two groups of offspring, the overall abundance of short-chain fatty acid producing bacteria in IO group was not increased. According to the hygiene hypothesis [45], exposure to pathogens early in life actually facilitates the training and development of the human immune system. The IO group in this experiment produced a higher total ASV number, suggesting that they have more exposure to microorganisms that influence immune development and thus reducing the risk of allergic disease. The experiment by Holly Bachus et al. [46] showed that higher doses of LPS prevented infants from developing allergic Th2 cell responses, revealing a potential mechanism that supports the hygiene hypothesis. However, it has also been found that LPS synthesized by different gut microorganisms have different promoting or inhibiting effects on the immune system [47].

**Fig 7:**
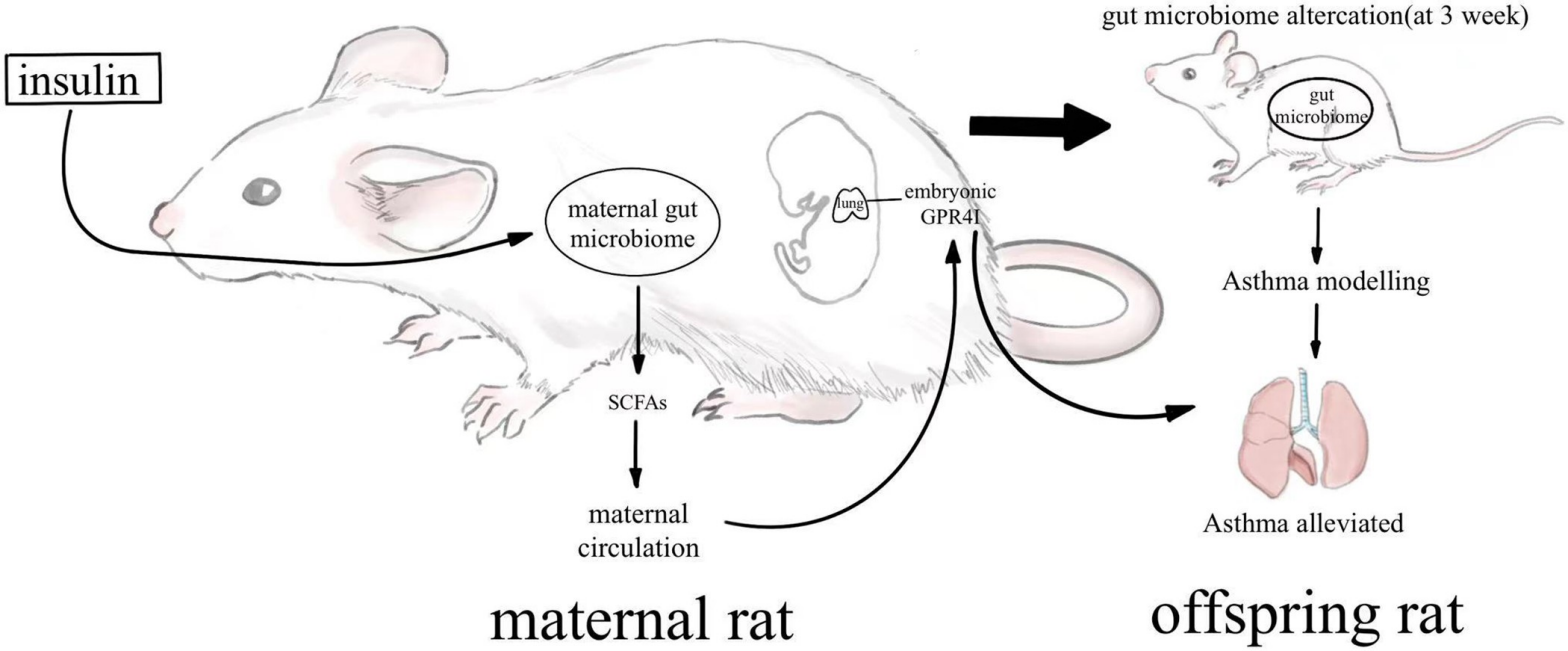
The influence of maternal inulin diet on gut microbiome and GPR41 expression of offspring.

Oral administration of inulin alters the composition of the intestinal flora, and it also increases the levels of the flora metabolites SCFAs [11]. SCFAs are the ligands of GPR41 and GPR43, and those SCFAs in maternal gut are also capable of infiltrating the placental barrier and alleviating offspring’s susceptibility to disease by affecting GPR41 and GPR43 [48]. Therefore, we examined the intestinal microbiome of both groups of pregnant rats at E18.5 and the GPR41 and GPR43 levels in the lung tissue of their offspring. Furthermore, the SCFAs content in the feces of the maternal rats fed inulin was lower than that of the rats fed a normal diet. However, the relative expression of GPR41 and GPR43 in the lung tissues of the offspring of both groups were higher in the lung tissues of the offspring of maternal rats fed inulin than in the offspring of pregnant rats on a normal diet, in which GPR41 being statistically significant. GPR41 is a short-chain fatty acid receptor activated mainly by acetate, propionate, and butyrate and coupled to Gi/o proteins that inhibit cAMP production and promote phosphorylation of ERK1/2 [49]. It has been shown that consumption of soluble fiber increases GPR43 and GPR41 gene expression in sputum of asthma patients and improves airway inflammation [50]. Inulin, a soluble dietary fiber, increases serum levels of acetic, propionic, and butyric acids [51, 52]. Propionate, one of its products, is dependent on GPR41 but not GPR43 for its protective effect against airway allergic inflammation [53]. A study in 2020 reported that embryos can perceive the change of the short-chain fatty acids from maternal origin through blood circulation [20]. These studies might be able to explain the paradoxical results between the levels of short-chain fatty acid and high expression of GPR41 in offspring(Fig 7). The maternal short chain fatty acid might play a greater role in this progression. Akihito had reported that the maternal microbiome has a great impact on the development of the offspring’s immune system. The offspring of high fiber diet fed mice exhibited higher autoimmune regulator (Aire) expression, a transcription factor expressed in the thymic microenvironment, suggesting SCFAs promote thymic Treg differentiation through increased Aire expression [54].

## Conclusions

The present study demonstrates for the first time that consumption of inulin during pregnancy altered maternal intestinal microbiome composition, with a significant increase in SCFAs-producing bacteria and attenuates the asthmatic inflammatory response in offspring. Although the specific mechanisms were not explored further due to time constraints and external force majeure limitations, we speculated on the possible mechanisms based on the current results and literature reports. SCFAs produced by maternal high inulin fed might pass through the placental barrier and modulate the differentiation of T cells to improve TH1/TH2 imbalance, thereby reducing inflammation in the offspring lung. We will subsequently validate the mechanism of action of GPR41 in regulating asthma inflammation by using GPR41 modulators (agonists or antagonists) and verifying the alteration of TH1/TH2 cells in lung tissue.

## Supporting information

S1 Table Formula of regular feed. (DOCX)

## References

[1] Boonpiyathad T, Sözener ZC, Satitsuksanoa P, Akdis CA. Immunologic mechanisms in asthma. Semin Immunol. 2019;46:101333. Epub 2019/11/11. doi: 10.1016/j.smim.2019.101333 . 31703832

[2] Trikamjee T, Comberiati P, Peter J. Pediatric asthma in developing countries: challenges and future directions. Curr Opin Allergy Clin Immunol. 2022;22(2):80–5. Epub 2022/02/25. doi: 10.1097/ACI.0000000000000806 . 35197428

[3] Ellwood P, Asher MI, García-Marcos L, Williams H, Keil U, Robertson C, et al. Do fast foods cause asthma, rhinoconjunctivitis and eczema? Global findings from the International Study of Asthma and Allergies in Childhood (ISAAC) phase three. Thorax. 2013;68(4):351–60. Epub 2013/01/16. doi: 10.1136/thoraxjnl-2012-202285 . 23319429

[4] Alwarith J, Kahleova H, Crosby L, Brooks A, Brandon L, Levin SM, et al. The role of nutrition in asthma prevention and treatment. Nutr Rev. 2020;78(11):928–38. Epub 2020/03/14. doi: 10.1093/nutrit/nuaa005 ; PubMed Central PMCID: PMC7550896. 32167552 PMC7550896

[5] Herbst T, Sichelstiel A, Schär C, Yadava K, Bürki K, Cahenzli J, et al. Dysregulation of allergic airway inflammation in the absence of microbial colonization. Am J Respir Crit Care Med. 2011;184(2):198–205. Epub 2011/04/08. doi: 10.1164/rccm.201010-1574OC . 21471101

[6] Verstegen REM, Kostadinova AI, Merenciana Z, Garssen J, Folkerts G, Hendriks RW, et al. Dietary Fibers: Effects, Underlying Mechanisms and Possible Role in Allergic Asthma Management. Nutrients. 2021;13(11). Epub 2021/11/28. doi: 10.3390/nu13114153 ; PubMed Central PMCID: PMC8621630. 34836408 PMC8621630

[7] Gupta N, Jangid AK, Pooja D, Kulhari H. Inulin: A novel and stretchy polysaccharide tool for biomedical and nutritional applications. Int J Biol Macromol. 2019;132:852–63. Epub 2019/03/31. doi: 10.1016/j.ijbiomac.2019.03.188 . 30926495

[8] Song X, Zhong L, Lyu N, Liu F, Li B, Hao Y, et al. Inulin Can Alleviate Metabolism Disorders in ob/ob Mice by Partially Restoring Leptin-related Pathways Mediated by Gut Microbiota. Genomics Proteomics Bioinformatics. 2019;17(1):64–75. Epub 2019/04/27. doi: 10.1016/j.gpb.2019.03.001 ; PubMed Central PMCID: PMC6520907. 31026583 PMC6520907

[9] Holscher HD, Bauer LL, Gourineni V, Pelkman CL, Fahey GC Jr., Swanson KS. Agave Inulin Supplementation Affects the Fecal Microbiota of Healthy Adults Participating in a Randomized, Double-Blind, Placebo-Controlled, Crossover Trial. J Nutr. 2015;145(9):2025–32. Epub 2015/07/24. doi: 10.3945/jn.115.217331 . 26203099

[10] Fuhren J, Schwalbe M, Boekhorst J, Rösch C, Schols HA, Kleerebezem M. Dietary calcium phosphate strongly impacts gut microbiome changes elicited by inulin and galacto-oligosaccharides consumption. Microbiome. 2021;9(1):218. Epub 2021/11/05. doi: 10.1186/s40168-021-01148-0 ; PubMed Central PMCID: PMC8567720. 34732247 PMC8567720

[11] Han K, Nam J, Xu J, Sun X, Huang X, Animasahun O, et al. Generation of systemic antitumour immunity via the in situ modulation of the gut microbiome by an orally administered inulin gel. Nat Biomed Eng. 2021;5(11):1377–88. Epub 2021/06/26. doi: 10.1038/s41551-021-00749-2 ; PubMed Central PMCID: PMC8595497. 34168321 PMC8595497

[12] McLoughlin R, Berthon BS, Rogers GB, Baines KJ, Leong LEX, Gibson PG, et al. Soluble fibre supplementation with and without a probiotic in adults with asthma: A 7-day randomised, double blind, three way cross-over trial. EBioMedicine. 2019;46:473–85. Epub 2019/08/04. doi: 10.1016/j.ebiom.2019.07.048 ; PubMed Central PMCID: PMC6712277. 31375426 PMC6712277

[13] Dalile B, Van Oudenhove L, Vervliet B, Verbeke K. The role of short-chain fatty acids in microbiota-gut-brain communication. Nat Rev Gastroenterol Hepatol. 2019;16(8):461–78. Epub 2019/05/28. doi: 10.1038/s41575-019-0157-3 . 31123355

[14] Pouteau E, Nguyen P, Ballèvre O, Krempf M. Production rates and metabolism of short-chain fatty acids in the colon and whole body using stable isotopes. Proc Nutr Soc. 2003;62(1):87–93. Epub 2003/05/13. doi: 10.1079/PNS2003208 . 12740063

[15] Layden B, Angueira A, Brodsky M, Durai V, Lowe W. Short chain fatty acids and their receptors: new metabolic targets. Translational research: the journal of laboratory and clinical medicine. 2013;161(3):131–40. doi: 10.1016/j.trsl.2012.10.007 23146568

[16] Yao Y, Cai X, Fei W, Ye Y, Zhao M, Zheng C. The role of short-chain fatty acids in immunity, inflammation and metabolism. Critical reviews in food science and nutrition. 2022;62(1):1–12. doi: 10.1080/10408398.2020.1854675 . 33261516

[17] Richards JL, Yap YA, McLeod KH, Mackay CR, Mariño E. Dietary metabolites and the gut microbiota: an alternative approach to control inflammatory and autoimmune diseases. Clin Transl Immunology. 2016;5(5):e82. Epub 2016/06/29. doi: 10.1038/cti.2016.29 ; PubMed Central PMCID: PMC4910123. 27350881 PMC4910123

[18] Yip W, Hughes MR, Li Y, Cait A, Hirst M, Mohn WW, et al. Butyrate Shapes Immune Cell Fate and Function in Allergic Asthma. Front Immunol. 2021;12:628453. Epub 2021/03/05. doi: 10.3389/fimmu.2021.628453 ; PubMed Central PMCID: PMC7917140. 33659009 PMC7917140

[19] Maslowski KM, Vieira AT, Ng A, Kranich J, Sierro F, Yu D, et al. Regulation of inflammatory responses by gut microbiota and chemoattractant receptor GPR43. Nature. 2009;461(7268):1282–6. Epub 2009/10/30. doi: 10.1038/nature08530 ; PubMed Central PMCID: PMC3256734. 19865172 PMC3256734

[20] Trompette A, Gollwitzer ES, Yadava K, Sichelstiel AK, Sprenger N, Ngom-Bru C, et al. Gut microbiota metabolism of dietary fiber influences allergic airway disease and hematopoiesis. Nat Med. 2014;20(2):159–66. Epub 2014/01/07. doi: 10.1038/nm.3444 . 24390308

[21] Mizuta K, Sasaki H, Zhang Y, Matoba A, Emala CW Sr. The short-chain free fatty acid receptor FFAR3 is expressed and potentiates contraction in human airway smooth muscle. Am J Physiol Lung Cell Mol Physiol. 2020;318(6):L1248–l60. Epub 2020/03/27. doi: 10.1152/ajplung.00357.2019 ; PubMed Central PMCID: PMC7347267. 32209026 PMC7347267

[22] Saglani S, Bush A. The early-life origins of asthma. Curr Opin Allergy Clin Immunol. 2007;7(1):83–90. Epub 2007/01/16. doi: 10.1097/ACI.0b013e32801297e6 . 17218816

[23] Kumar R. Prenatal factors and the development of asthma. Curr Opin Pediatr. 2008;20(6):682–7. Epub 2008/11/14. doi: 10.1097/MOP.0b013e3283154f26 . 19005336

[24] Vuong HE, Pronovost GN, Williams DW, Coley EJL, Siegler EL, Qiu A, et al. The maternal microbiome modulates fetal neurodevelopment in mice. Nature. 2020;586(7828):281–6. Epub 2020/09/25. doi: 10.1038/s41586-020-2745-3 ; PubMed Central PMCID: PMC7554197. 32968276 PMC7554197

[25] McDonald B, McCoy KD. Maternal microbiota in pregnancy and early life. Science. 2019;365(6457):984–5. Epub 2019/09/07. doi: 10.1126/science.aay0618 . 31488675

[26] Pronovost GN, Hsiao EY. Perinatal Interactions between the Microbiome, Immunity, and Neurodevelopment. Immunity. 2019;50(1):18–36. Epub 2019/01/17. doi: 10.1016/j.immuni.2018.11.016 ; PubMed Central PMCID: PMC6447295. 30650376 PMC6447295

[27] Gomez de Agüero M, Ganal-Vonarburg SC, Fuhrer T, Rupp S, Uchimura Y, Li H, et al. The maternal microbiota drives early postnatal innate immune development. Science. 2016;351(6279):1296–302. Epub 2016/03/19. doi: 10.1126/science.aad2571 . 26989247

[28] Russell SL, Gold MJ, Willing BP, Thorson L, McNagny KM, Finlay BB. Perinatal antibiotic treatment affects murine microbiota, immune responses and allergic asthma. Gut Microbes. 2013;4(2):158–64. Epub 2013/01/22. doi: 10.4161/gmic.23567 ; PubMed Central PMCID: PMC3595077. 23333861 PMC3595077

[29] Ferguson J. Maternal microbial molecules affect offspring health. Science. 2020;367(6481):978–9. Epub 2020/02/29. doi: 10.1126/science.aba7673 . 32108100

[30] Thorburn AN, McKenzie CI, Shen S, Stanley D, Macia L, Mason LJ, et al. Evidence that asthma is a developmental origin disease influenced by maternal diet and bacterial metabolites. Nat Commun. 2015;6:7320. Epub 2015/06/24. doi: 10.1038/ncomms8320 . 26102221

[31] O’Keefe SJ. The association between dietary fibre deficiency and high-income lifestyle-associated diseases: Burkitt’s hypothesis revisited. Lancet Gastroenterol Hepatol. 2019;4(12):984–96. Epub 2019/11/08. doi: 10.1016/S2468-1253(19)30257-2 ; PubMed Central PMCID: PMC6944853. 31696832 PMC6944853

[32] Gor DO, Rose NR, Greenspan NS. TH1-TH2: a procrustean paradigm. Nat Immunol. 2003;4(6):503–5. Epub 2003/05/30. doi: 10.1038/ni0603-503 . 12774069

[33] Le Bastard Q, Chapelet G, Javaudin F, Lepelletier D, Batard E, Montassier E. The effects of inulin on gut microbial composition: a systematic review of evidence from human studies. Eur J Clin Microbiol Infect Dis. 2020;39(3):403–13. Epub 2019/11/11. doi: 10.1007/s10096-019-03721-w . 31707507

[34] Fukuda S, Toh H, Hase K, Oshima K, Nakanishi Y, Yoshimura K, et al. Bifidobacteria can protect from enteropathogenic infection through production of acetate. Nature. 2011;469(7331):543–7. Epub 2011/01/29. doi: 10.1038/nature09646 . 21270894

[35] Seganfredo FB, Blume CA, Moehlecke M, Giongo A, Casagrande DS, Spolidoro JVN, et al. Weight-loss interventions and gut microbiota changes in overweight and obese patients: a systematic review. Obes Rev. 2017;18(8):832–51. Epub 2017/05/20. doi: 10.1111/obr.12541 . 28524627

[36] Benítez-Páez A, Gómez Del Pugar EM, López-Almela I, Moya-Pérez Á, Codoñer-Franch P, Sanz Y. Depletion of Blautia Species in the Microbiota of Obese Children Relates to Intestinal Inflammation and Metabolic Phenotype Worsening. mSystems. 2020;5(2). Epub 2020/03/27. doi: 10.1128/mSystems.00857-19 ; PubMed Central PMCID: PMC7093825. 32209719 PMC7093825

[37] Franke T, Deppenmeier U. Physiology and central carbon metabolism of the gut bacterium Prevotella copri. Mol Microbiol. 2018;109(4):528–40. Epub 2018/07/12. doi: 10.1111/mmi.14058 . 29995973

[38] Cummings JH, Pomare EW, Branch WJ, Naylor CP, Macfarlane GT. Short chain fatty acids in human large intestine, portal, hepatic and venous blood. Gut. 1987;28(10):1221–7. Epub 1987/10/01. doi: 10.1136/gut.28.10.1221 ; PubMed Central PMCID: PMC1433442. 3678950 PMC1433442

[39] Vuillermin PJ, O’Hely M, Collier F, Allen KJ, Tang MLK, Harrison LC, et al. Maternal carriage of Prevotella during pregnancy associates with protection against food allergy in the offspring. Nat Commun. 2020;11(1):1452. Epub 2020/03/27. doi: 10.1038/s41467-020-14552-1 32210229 PMC7093478

[40] Ziętek M, Celewicz Z, Szczuko M. Short-Chain Fatty Acids, Maternal Microbiota and Metabolism in Pregnancy. Nutrients. 2021 Apr 9;13(4):1244. doi: 10.3390/nu13041244 33918804 PMC8069164

[41] Nettleton JE, Cho NA, Klancic T, Nicolucci AC, Shearer Jane, et al. Maternal low-dose aspartame and stevia consumption with an obesogenic diet alters metabolism, gut microbiota and mesolimbic reward system in rat dams and their offspring. Gut. 2020;69(10):1807–1817. doi: 10.1136/gutjnl-2018-317505 31996393 PMC7497576

[42] Lundgren SN, Madan JC, Emond JA, Morrison HG, Christensen BC, Karagas MR, et al. Maternal diet during pregnancy is related with the infant stool microbiome in a delivery mode-dependent manner. Microbiome. 2018;6(1):109. Epub 2018/07/06. doi: 10.1186/s40168-018-0490-8 ; PubMed Central PMCID: PMC6033232. 29973274 PMC6033232

[43] Qin R, Wang J, Chao C, Yu J, Copeland L, Wang S, et al. RS5 Produced More Butyric Acid through Regulating the Microbial Community of Human Gut Microbiota. J Agric Food Chem. 2021;69(10):3209–18. Epub 2021/02/26. doi: 10.1021/acs.jafc.0c08187 33630575

[44] Wu F, Guo X, Zhang J, Zhang M, Ou Z, Peng Y. Phascolarctobacterium faecium abundant colonization in human gastrointestinal tract. Exp Ther Med. 2017;14(4):3122–6. Epub 2017/09/16. doi: 10.3892/etm.2017.4878 ; PubMed Central PMCID: PMC5585883. 28912861 PMC5585883

[45] Strachan DP. Hay fever, hygiene, and household size. Bmj. 1989;299(6710):1259–60. Epub 1989/11/18. doi: 10.1136/bmj.299.6710.1259 ; PubMed Central PMCID: PMC1838109. 2513902 PMC1838109

[46] Bachus H, Kaur K, Papillion AM, Marquez-Lago TT, Yu Z, Ballesteros-Tato A, et al. Impaired Tumor-Necrosis-Factor-α-driven Dendritic Cell Activation Limits Lipopolysaccharide-Induced Protection from Allergic Inflammation in Infants. Immunity. 2019;50(1):225–40.e4. Epub 2019/01/13. doi: 10.1016/j.immuni.2018.11.012 30635238 PMC6335154

[47] Vatanen T, Kostic AD, d’Hennezel E, Siljander H, Franzosa EA, Yassour M, et al. Variation in Microbiome LPS Immunogenicity Contributes to Autoimmunity in Humans. Cell. 2016;165(6):1551. Epub 2016/06/04. doi: 10.1016/j.cell.2016.05.056 . 27259157

[48] Chang PV, Hao L, Offermanns S, Medzhitov R. The microbial metabolite butyrate regulates intestinal macrophage function via histone deacetylase inhibition. Proc Natl Acad Sci U S A. 2014;111(6):2247–52. Epub 2014/01/07. doi: 10.1073/pnas.1322269111 ; PubMed Central PMCID: PMC3926023. 24390544 PMC3926023

[49] Kimura I, Ichimura A, Ohue-Kitano R, Igarashi M. Free Fatty Acid Receptors in Health and Disease. Physiological reviews. 2020;100(1):171–210. doi: 10.1152/physrev.00041.2018 . 31487233

[50] Halnes I, Baines K, Berthon B, MacDonald-Wicks L, Gibson P, Wood L. Soluble Fibre Meal Challenge Reduces Airway Inflammation and Expression of GPR43 and GPR41 in Asthma. Nutrients. 2017;9(1). doi: 10.3390/nu9010057 . 28075383 PMC5295101

[51] Tarini J, Wolever T. The fermentable fibre inulin increases postprandial serum short-chain fatty acids and reduces free-fatty acids and ghrelin in healthy subjects. Applied physiology, nutrition, and metabolism = Physiologie appliquee, nutrition et metabolisme. 2010;35(1):9–16. doi: 10.1139/H09-119 . 20130660

[52] Gill S, Pop M, Deboy R, Eckburg P, Turnbaugh P, Samuel B, et al. Metagenomic analysis of the human distal gut microbiome. Science (New York, NY). 2006;312(5778):1355–9. doi: 10.1126/science.1124234 . 16741115 PMC3027896

[53] Kimura I, Miyamoto J, Ohue-Kitano R, Watanabe K, Yamada T, Onuki M, et al. Maternal gut microbiota in pregnancy influences offspring metabolic phenotype in mice. Science (New York, NY). 2020;367(6481). doi: 10.1126/science.aaw8429 . 32108090

[54] Nakajima Akihito, Kaga Naoko, Yumiko Nakanishi; Hiroshi Ohno, Junki Miyamoto, et al. Maternal High Fiber Diet during Pregnancy and Lactation Influences Regulatory T Cell Differentiation in Offspring in Mice. The Journal of immunology, 2017 Nov 15;199(10):3516–3524. doi: 10.4049/jimmunol.1700248 29021375

