# Supplementary material for: Inulin alleviates offspring asthma by altering maternal intestinal microbiome composition to increase short-chain fatty acids
Source: PLoS One. 2023 Apr 4;18(4):e0283105. doi: 10.1371/journal.pone.0283105 (PMC10072493; doi:10.1371/journal.pone.0283105)
Supplement: S1 Table — (DOCX) [file pone.0283105.s001.docx]

**S1 Table：Formula of Regular feed**

| **Ingredient g/kg** | **HF (5%)** |
| --- | --- |
| Casein | 200 |
| L-Cystine | 3 |
| Sucrose | 100 |
| Cornstarch | 397 |
| Dyetrose | 132 |
| Soybean Oil | 70 |
| t-Butylhydroquinone | 0.014 |
| Cellulose | 50 |
| Mineral Mix #210025 | 35 |
| Vitamin Mix # 310025 | 10 |
| Choline Bitartrate | 2.5 |
| Calories (kcal/kg) | 2860 |
